# Distinct Profiles of Effector Cytokines Mark the Different Phases of Crohn’s Disease

**DOI:** 10.1371/journal.pone.0054562

**Published:** 2013-01-17

**Authors:** Francesca Zorzi, Ivan Monteleone, Massimiliano Sarra, Emma Calabrese, Irene Marafini, Micaela Cretella, Silvia Sedda, Livia Biancone, Francesco Pallone, Giovanni Monteleone

**Affiliations:** Department of Systems Medicine, University of Rome “Tor Vergata”, Rome, Italy; INSERM, France

## Abstract

**Objective:**

Crohn’s Disease (CD)-associated inflammation is supposed to be driven by T helper (Th)1/Th17 cell-derived cytokines, even though there is evidence that the mucosal profile of cytokine may vary with the evolution of the disease. We aimed at comparing the pattern of effector cytokines in early and established lesions of CD.

**Design:**

Mucosal samples were taken from the neo-terminal ileum of CD patients undergoing ileocolonic resection, with (early lesions) or without post-operative recurrence, and terminal ileum of CD patients with long-standing disease undergoing intestinal resection (established lesions). Inflammatory cell infiltrate was examined by immunofluorescence and cytokine expression was analysed by real-time PCR, flow-cytometry and ELISA.

**Results:**

Before the appearance of endoscopic lesions, the mucosa of the neo-terminal ileum contained high number of T cells and macrophages, elevated levels of Th1-related cytokines and TNF-α and slightly increased IL-17A expression. Transition from this stage to endoscopic recurrence was marked by abundance of Th1 cytokines, marked increase in IL-17A, and induction of IL-6 and IL-23, two cytokines involved in the control of Th17 cell responses. In samples with established lesions, there was a mixed Th1/Th17 response with no TNF-α induction. Expression of IL-4 and IL-5 was up-regulated in both early and established lesions even though the fraction of IL-4-producing cells was lower than that of cells producing either interferon-γ or IL-17A.

**Conclusions:**

Distinct mucosal profiles of cytokines are produced during the different phases of CD. A better understanding of the cytokines temporally regulated in CD tissue could help optimize therapeutic interventions in CD.

## Introduction

Crohn’s Disease (CD) is a chronic relapsing inflammatory disorder of the gastrointestinal tract. The etiopathogenesis of CD is not fully understood, but genetic and environmental factors interact to promote an excessive and poorly controlled mucosal inflammatory response directed against components of the gut microflora. [1]–[2] Functional abnormalities of many components of the immune system can be seen in the damaged gut of CD patients, but hyperactivity of T cells with excessive production of inflammatory cytokines is believed to be one of the major immunological hallmarks of this disorder. CD-associated destructive immune response is polarized along the T helper (Th)1 cell pathway, as indicated by the demonstration that mucosal CD4+T cells produce large quantities of interferon (IFN)-γ [3] and over-express T-bet, a transcription factor necessary for driving and sustaining Th1 cell responses. [4] CD tissue also contains high interleukin (IL)-12, [5] the major Th1-inducing factor in humans, [6] and IL-18, a cytokine that expands Th1 cell responses. [7] Despite these observations and the demonstration that Th1-type cytokines are pro-inflammatory in murine models of CD, [8] blockade of IFN-γ with a neutralizing antibody (i.e. Fontolizumab) was not beneficial in CD patients. [9]–[10] These disappointing results could rely on the fact that the CD-associated tissue lesions are driven by multiple and disconnected inflammatory pathways, which are not fully inhibited by Fontolizumab. Indeed, the inflamed gut of CD patients is also massively infiltrated with a distinct subset of Th cells, termed Th17 cells, which over-express the transcription factors retinoic acid-related orphan receptor (ROR)-γt and RORα, produce IL-17A, IL-17F, IL-21, IL-22, and IL-26, and are negatively regulated by IFN-γ. [11]–[14] Even the administration of a neutralizing IL-17A antibody was not effective in CD, thus confirming the complexity of the tissue-damaging immunoinflammatory events in CD. [15] Data emerging from recent studies raise the possibility that the mucosal cytokine profile in CD is not stable and may vary with the course of the disease thus contributing to the lack of therapeutic response to cytokine blockers. For example, analysis of T cell-derived cytokines in supernatants of T cell clones derived from intestinal biopsies of children with CD and stimulated with IL-12 revealed that IFN-γ levels were markedly elevated in patients with first attack of CD but not in those with established lesions. [16] Moreover some of the IL-17A-producing cells infiltrating CD tissue co-express IFN-γ and can lack IL-17A and be converted into Th1 cells following stimulation with IL-12. [17] In CD, deviation from a Th17 to a Th1 phenotype could be favoured not only by high IL-12 but also defects in TGF-β1 activity due to high Smad7, an intracellular inhibitor of TGF-β1-driven signalling, because TGF-β1 is needed to sustain IL-17A production by Th17 cells [18]–[21].

Altogether these observations indicate that better understanding of the cytokines temporally regulated in CD tissue is needed to identify optimal targets for therapeutic interventions. The aim of this study was to assess whether, in CD, the distribution patterns of cytokines in early lesions (i.e. lesions in the neo-terminal ileum of CD patients following a curative ileocolonic resection) differs from that seen in established/late lesions (lesions requiring surgery).

## Materials and Methods

### Ethics Statement

Each patient who took part in the study gave written informed consent and the study was approved by the local ethics committee (Tor Vergata University Hospital, Rome).

### Patients and Samples

Mucosal samples were taken from resection specimens of 9 CD patients [4 male; median age 51 (21–67) years, median disease duration 144 (36–312) months] undergoing resection for a chronically active disease poorly responsive to medical treatment. In all these patients, lesions (herein termed late/established CD) were confined to the terminal ileum. At the time of surgery, all patients were on steroids; 2 of them were taking simultaneously azathioprine, while 4 had received at least 3 infusions of anti TNF-α in the previous months. Ileocolonoscopy was performed 6 (n = 5) or 12 (n = 4) months after the intestinal resection for ascertaining the presence of post-operative recurrence and mucosal biopsies were taken from the neo-terminal ileum for evaluating cytokine expression. Ileal biopsies were also collected from the neo-terminal ileum of 10 additional CD patients [10 male; median age 34 (22–61) years], who underwent ileo-colonoscopy for assessing the occurrence of recurrence 6 (n = 5) or 12 (n = 5) months after ileo-colectomy and ileocolonic anastomosis. In this group of patients, indications for surgery were active CD poorly responsive to medical treatment. Timing of ileocolonoscopy was selected taking into account the clinical activity of disease and past history of severe disease. In all the 19 patients considered for the study, mesalamine was started immediately after surgery and no other drug was prescribed for preventing recurrence until the patients underwent ileocolonoscopy. Overall, 5 out of 19 (26,3%) patients examined for the presence of post-operative recurrence had a clinically active disease (CDAI>150). Endoscopic recurrence was evaluated during ileocolonoscopy and graded according to the Rutgeerts’s score (0: no lesions; 1: less than 5 aphthous lesions; 2: more than 5 aphthous lesions with normal mucosa between the lesions, or skip areas of larger lesions, or lesions confined to the ileocolonic anastomotic lining; 3: diffuse aphthous ileitis with diffusely inflamed mucosa; and 4: diffuse ileal inflammation with larger ulcers, nodules, or narrowing. Hyperaemia and oedema alone were not considered as signs of recurrence). [22] Ileal biopsies were collected from the neo-terminal ileum, 10–30 cm above the anastomosis.

Ileal biopsies were also taken from 5 healthy controls who underwent ileocolonoscopy for irritable bowel syndrome. No endoscopic lesions were found in the control group, and the ileal mucosa was histologically normal.

### Immunofluorescence

Frozen sections of mucosal samples were stained with monoclonal mouse anti-human CD3 (1∶100 final dilution; Santa Cruz Biotechnology, DBA, Milan, Italy) and monoclonal mouse anti-human CD68 (1∶200 final dilution; Dako, Glostrup, Denmark) followed by incubation with a highly sensitive biotinylated secondary Ab (Dako) and a Tyramide Signal Amplification Kit (PerkinElmer, Waltham, MA). CD3-positive cells and CD68-positive cells were counted and expressed as numbers of cells×high power field and 5 high power fields were subsequently counted in each slide.

### Lamina Propria Mononuclear Cell Isolation

All reagents were from Sigma-Aldrich (Milan, Italy) unless specified. Lamina propria mononuclear cells (LPMC) were isolated from ileal biopsies and intestinal resection specimens of CD patients and normal controls as described elsewhere. [5] LPMC were suspended in RPMI 1640 medium, supplemented with 10% inactivated fetal bovine serum (FBS), penicillin (P) (100 U/ml), and streptomycin (S) (100 μg/ml) (Life Technologies-GibcoCRL, Milan, Italy). LPMC were used to assess cytokine expression by flow cytometry.

### RNA Extraction, cDNA Preparation, and Real-time PCR

RNA was extracted from fresh mucosal samples of CD patients and normal controls using Trizol reagent according to the manufacturer’s instructions (Invitrogen, Carlsbad, CA). A constant amount of RNA (1 mg per sample) was reverse-transcribed into cDNA, and this was amplified using a sybergreen-based PCR (Bio-Rad, Hercules, CA). PCR conditions were as follows: denaturation 1 min at 95°C, annealing 30 s at 61°C for IL-17A and IL-6; 58°C for IFN-γ, IL-21, IL-13 and IL-23p19; 62°C for TNF-α and IL-5, and 60°C for β-actin followed by 30 s extension at 72°C. Primer sequences were as follows: IL-17A forward 5′-ACTACAACCGATCCACCTCAC-3′, reverse 5′-ACTTTGCCTCCCAGATCACAG-3′; IL-6 forward 5′-CCACTCACCTCTTCAGAACG-3′, reverse 5′-GCCTCTTTGCTGCTTTCACAC-3′; IFN-γ forward 5′-TGGAGACCATCAAGGAAGAC-3′, reverse 5′-GCGTTGGACATTCAAGTCAG-3′; IL-21 forward 5′-GGAGAGGATTGTCATCTGTC-3′, reverse 5′-CACAGTTTGTCTCTACATCTTC-3′; IL-13 forward 5′-ACGGTCATTGCTCTCACTTG-3′, reverse 5′-GTCAGGTTGATGCTCCATAC-3′; IL-5 forward 5′-GATAGCCAATGAGACTCTGAGG-3′, reverse 5′-GCACAGTTTGACTCTCCAGTG-3′; IL-23p19 forward 5′-GGGACACATGGATCTAAGAG-3, reverse 5′-GCAAGCAGAACTGACTGTTG-3; TNF-α forward 5′-AGGCGGTGCTTGTTCCTCAG-3′, reverse 5′-GGCTACAGGCTTGTCACTCG-3′. IL-4, IL-12p40 and IL-12p35 were evaluated using commercially available TaqMan probes (Applied Biosystems, Foster City, CA). β-actin (forward 5′-AAGATGACCCAGATCATGTTTGAGACC-′3 and reverse 5′-AGCCAGTCCAGACGCAGGAT-′3) was used as a housekeeping gene. Gene expression was calculated using the ΔΔCt algorithm.

### Flow-cytometry Analysis

LPMC were seeded in 96-well U-bottom culture dishes and stimulated with PMA (10 ng/mL), ionomycin (1 µg/mL), and brefeldinA (10 µg/mL; eBioscience, San Diego, CA). After 5 h, cells were stained with the following Abs: anti–CD3-PerCP (1∶50, final dilution, BD Biosciences, San Jose, CA) and fixed with 1% formaldehyde for 20′. Subsequently cells were permeabilized with 0.5% saponin in 1% BSA FACS buffer and stained with the following Abs: anti–IFN-γ–PE (1∶50, final dilution; BD Biosciences), anti–IL-17A–APC (1∶50, final dilution, eBioscience), anti-IL-4-allophycocyanin (1∶50 final dilution, Biolegend, San Diego, CA), anti-IL-21-PE(1∶50, final dilution, eBioscience). Appropriate isotype-matched controls from BD Biosciences were included in all of the experiments. Cells were analysed using a FACSCalibur cytometer and Cell-QuestPro software.

### Total Protein Extraction and Enzyme-linked Immunosorbent Assay (ELISA)

Intestinal mucosal samples were lysed on ice in buffer containing 10 mM HEPES (pH 7.9), 10 mM KCl, 0.1 mM EDTA, 0.2 mM EGTA, and 0.5% Nonidet P40, supplemented with 1 mM dithiothreitol, 10 mg ml^–1^aprotinin, 10 mg ml^–1^ leupeptin, 1 mM phenyl-methylsulfonyl fluoride, 1 mM Na3VO4, and 1 mM NaF. Lysates were clarified by centrifugation at 12,000 *g* for 30 min at 4°C. Extracts were analysed for IL-12 content using sensitive commercial ELISA kits (R&D Systems, Minneapolis, MN) according to the manufacturer’s instructions.

### Statistical Analysis

Statistical differences were assessed with the GraphPad Prism statistical PC program (GraphPad Software, San Diego, CA). Comparisons were made between each CD subgroup and normal controls, and in CD group between early and established lesions using the Mann-Whitney U test (for cytokine expression) and the Student t-test (for CD3- and CD68-infiltrates). A p value of less than 0.05 was considered statistically significant.

## Results

### Clinical and Endoscopic Data

No endoscopic recurrence was documented in 8 out of 19 (42%) patients. Of the remaining 11 patients with endoscopic recurrence, 6 had diffuse inflammation and large ulcers (i4 grade), 2 had diffuse aphthous ileitis (i3 grade) and 3 had more than 5 aphthous lesion with normal mucosa between the lesions (i2 grade). The 5 patients with CDAI >150 had endoscopic recurrence (i2-i4).

### CD3+ and CD68+ Cells Infiltrate the Neo-terminal Ileum of CD Patients Independently of the Presence of Endoscopic Recurrence

**Figure 1:**
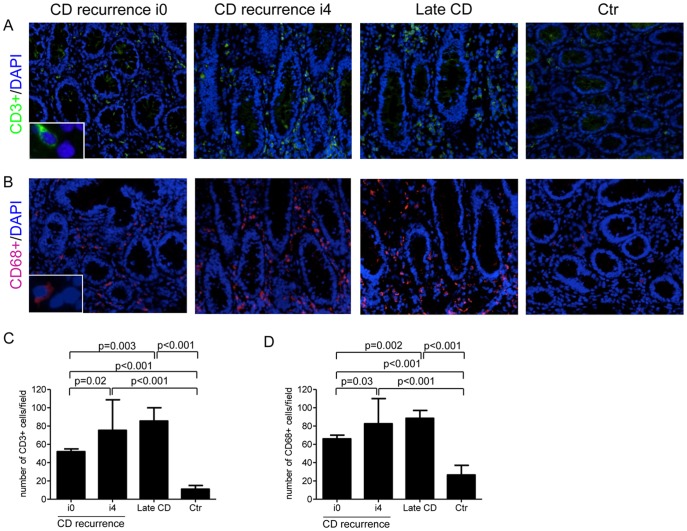
CD3 and CD68 positive cells accumulate in the neo-terminal ileum of Crohn’s disease patients. Representative immunofluorescence pictures of ileal sections of 1 CD patient with no evidence of endoscopic recurrence (i0), 1 CD patient with severe endoscopic recurrence (i4), 1 CD patient with established (late) lesion and 1normal control and stained with CD3+/DAPI (A) and CD68+/DAPI (B). Original magnification 100x. Insets in the left images show CD3 positive cells (A) and CD68 positive cells (B) at higher magnification (200x). C–D. Quantification of CD3+ and CD68+ cells in intestinal mucosa of 5 CD patients with no endoscopic recurrence (i0–i1), 5 CD patients with endoscopic recurrence (i2–i4), 5 CD patients with established lesions and 5 normal controls. Data are presented as mean values of positive cells per high power field ± SD of 5 independent experiments in which 5 sections per group were analyzed.

Following ileocolonic resection, the new CD lesions almost invariably develop in the previously unaffected mucosa of the neo-terminal ileum proximally to the ileocolonic anastomosis. [23] This post-operative state is therefore an ideal setting to investigate immunological events that drive the initial lesions of CD. To this end, we collected biopsies from CD patients with or without endoscopic recurrence and looked at the T cell and macrophage mucosal infiltration by immunofluorescence. The number of CD3+ cells was significantly higher in biopsies taken from the neo-terminal ileum of CD patients without endoscopic recurrence than in normal control biopsies and was further increased in biopsies taken from patients with endoscopic recurrence and in surgical specimens with established lesions, with no significant difference between these later two groups (Fig. 1A and C). Similarly, biopsies taken from the neo-terminal ileum of CD patients with no endoscopic recurrence contained more CD68+ cells than control biopsies (Fig. 1B and D). Moreover, CD68+ cells were more abundant in biopsies with endoscopic recurrence and in samples with established lesions than in biopsies without endoscopic lesions (Fig. 1B and D). These data indicate that, even in the absence of endoscopic lesions, the mucosa of the neo-terminal ileum of CD patients is markedly infiltrated with inflammatory cells.

### The Early Stage of CD Inflammation is Dominated by Th1 Cytokines while a Mixed Th1/Th17 Response is Seen in Areas with Early or Established Lesions

**Figure 2:**
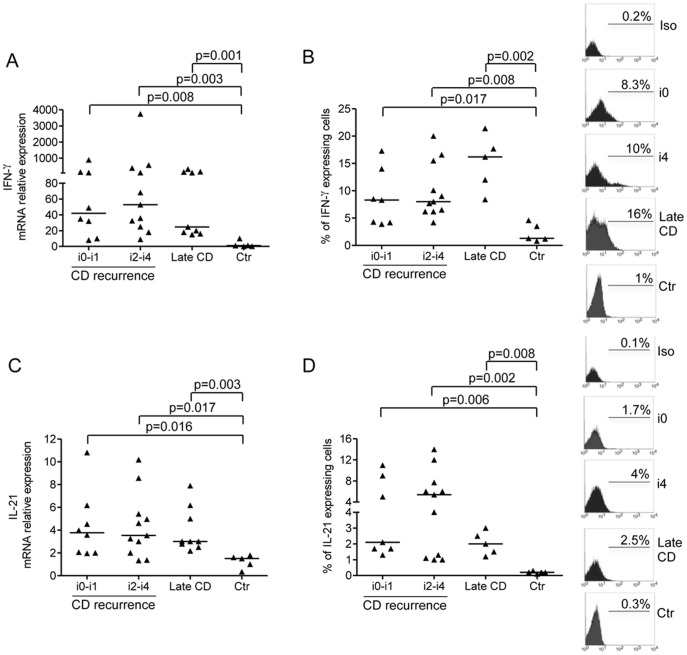
IFN-γ and IL-21 are up-regulated in the initial phase of CD inflammation. Transcripts for IFN-γ (A) and IL-21 (C) were analysed in ileal samples taken from CD patients with no endoscopic recurrence (i0–i1), CD patients with endoscopic recurrence (i2–i4), CD patients with established/late lesions and normal controls by real-time PCR and normalized to β-actin. Data indicate individual values of cytokines in single biopsies and horizontal bars represent the median value. B–D. Flow cytometry analysis of IFN-γ- and IL-21-producing cells in CD3+LPMC isolated from CD patients with no endoscopic recurrence (i0–i1), CD patients with endoscopic recurrence (i2–i4), CD patients with established/late lesions and normal controls. LPMC were gated on CD3+ cells and subsequently analysed for the expression of IFN-γ (B) and IL-21 (D). Data indicate individual values and horizontal bars represent the median value. Right insets: representative histograms of IFN-γ- and IL-21-producing CD3+cells in LPMC isolated from 1 CD patient with no endoscopic recurrence (i0), 1 CD patient with endoscopic recurrence (i4), 1 CD patient with established/late lesions and 1 normal control. Staining with a control IgG is also shown. Numbers above lines indicate the percentages of positive cells.

Next we examined various Th cell-related cytokines in CD and control samples by real-time PCR and flow-cytometry. Expression of IFN-γ transcripts was more pronounced in the biopsies taken from the neo-terminal ileum, either with or without endoscopic recurrence, and specimens with established lesions in comparison to control samples (Fig. 2A). Although there was variability in the content of transcripts among samples, no significant difference in terms of IFN-γ RNA was seen in mucosal samples taken from the 3 subgroups of CD patients (Fig. 2A). These data were confirmed by analysis of the percentages of IFN-γ-secreting cells in CD3+ LPMC samples isolated from biopsies and specimens of patients and controls (Fig. 2B). Since, in CD, IFN-γ-secreting cells produce IL-21, [24] we analysed IL-21in the same samples used for measuring IFN-γ. Up-regulation of IL-21 RNA and protein was seen in CD samples taken from the neo-terminal ileum, either with or without endoscopic recurrence, and established lesions (Fig. 2C–D).

**Figure 3:**
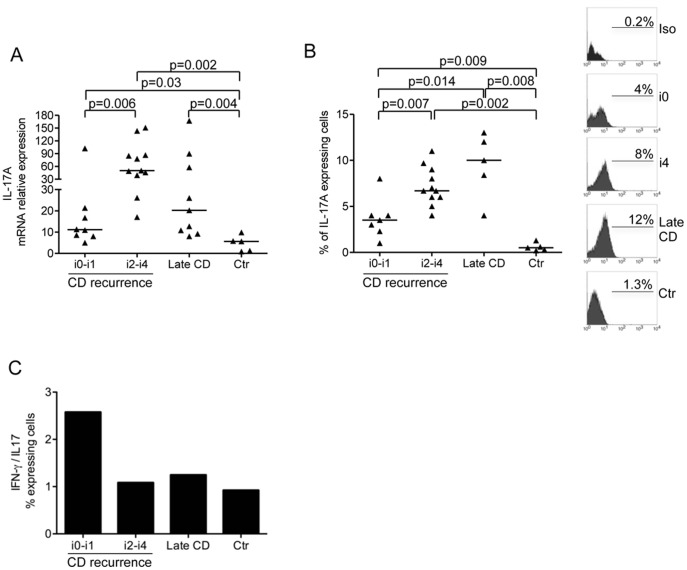
IL-17A is over-expressed in CD. Transcripts for IL-17A (A) were analysed in ileal samples taken from CD patients with no endoscopic recurrence (i0-i1), CD patients with endoscopic recurrence (i2-i4), CD patients with established/late lesions and normal controls by real-time PCR and normalized to β-actin. Data indicate individual values of IL-17A in single biopsies and horizontal bars represent the median value. B.Flow cytometry analysis of IL-17A-producing cells in CD3+LPMC isolated from CD patients with no endoscopic recurrence (i0-i1), CD patients with endoscopic recurrence (i2-i4), CD patients with established/late lesions and normal controls. LPMC were gated on CD3+ cells and subsequently analysed for the expression of IL-17A. Data indicate individual values and horizontal bars represent the median value. Right insets: representative histograms of IL-17A-producing CD3+cells in LPMC isolated from 1 CD patient with no endoscopic recurrence (i0), 1 CD patient with endoscopic recurrence (i4), 1 CD patient with established/late lesions and 1 normal control. Staining with a control IgG is also shown. Numbers above lines indicate the percentages of positive cells. C. Ratio between the percentages of IFN-γ-producing CD3+LPMC and IL-17A-producing CD3+ LPMC isolated from CD patients with no endoscopic recurrence (i0-i1), CD patients with endoscopic recurrence (i2-i4), CD patients with established/late lesions and normal controls.

CD-related inflammation is also associated with exaggerated Th17 cell response. [11]–[14] So, we next examined IL-17A in CD and control biopsies. Up-regulation of IL-17A RNA was observed in samples taken from the neo-terminal ileum, in presence or absence of endoscopic recurrence, and established lesions as compared to control patients (Fig. 3A). When analysis was restricted to biopsies taken from the neo-terminal ileum, it was evident that IL-17A RNA transcripts were significantly higher in samples with endoscopic recurrence (Fig. 3A). By flow-cytometry we then confirmed that IL-17A was over-expressed in the mucosal samples taken from the 3 subgroups of CD patients and that the percentages of IL-17A-secreting cells were significantly higher in the presence of macroscopically evident (both early and established) lesions (Fig. 3B). However, the percentage of cells in the neo-terminal ileum with no endoscopic lesions producing IFN-γ was nearly 3 times higher than the percentage of cells producing IL-17A, while these percentages were similar in areas with either early or established lesions (Fig. 3C).

Similar results were seen when cytokine RNA expression was performed in samples taken from 9 patients followed-up longitudinally before and after the intestinal resection (Tables 1–2).

**Table 1:** Cytokine expression in pre-operative (established) and post-operative ileal samples of Crohn’s disease patients.

	Established CD (n = 4)Median (range)	CD recurrence i0-i1 (n = 4)Median (range)
IFN-γ	18 (15–99)	76 (92–834)
IL-21	2,9 (2,2–6,2)	4,3 (3,6–10,8)
IL-17A	16,5 (10,9–26)	9 (5–10)
IL-4	7,7 (7,4–9)	4,5 (1–10,4)
IL-5	49,9 (30–76,2)	2,1 (1,8–13,8)
IL-13	23,6 (9–30)	12,4 (4,7–42)
TNF-α	5,2 (4,9–6,3)	20,1 (19,9–22,8)
IL-6	78,4 (14,3–294)	10,4 (7–13,8)

Post-operative samples were taken from areas with no endoscopic lesions.

**Table 2:** Cytokine expression in pre-operative (established) and post-operative ileal samples of Crohn’s disease patients.

	Established CD (n = 5)Median (range)	CD recurrence i2-i4 (n = 5)Median (range)
IFN-γ	140,8 (18–326)	68,3 (32,5–560)
IL-21	3 (2,5–7,9)	4,6 (2–10,2)
IL-17A	57,6 (10–168)	50 (45,7–143,4)
IL-4	225,5 (36,2–296)	38,3 (17,3–54,6)
IL-5	47,1 (24,2–191,7)	41,5 (29–127,5)
IL-13	30 (12–309)	32,7 (13,3–120)
TNF-α	7,8 (1,4–10,7)	21,6 (7,8–60,7)
IL-6	92,4 (32,6–288,9)	60,7 (13,8–342)

Post-operative samples were taken from areas with endoscopic recurrence.

### Over-expression of Th2-cytokines Occur in Both the Macroscopically Affected Neo-terminal Ileum and Established Lesions of CD Patients

**Figure 4:**
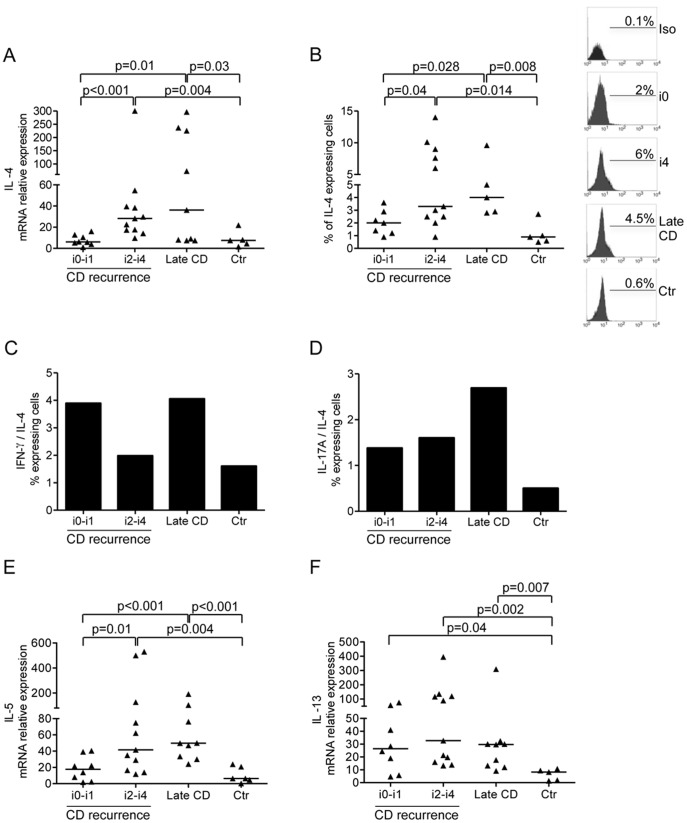
IL-4, IL-5 and IL-13 are up regulated in CD tissue with early and established lesions. Transcripts for IL-4 (A), IL-5 (E) and IL-13 (F) were analysed in ileal samples taken from CD patients with no endoscopic recurrence (i0–i1), CD patients with endoscopic recurrence (i2–i4), CD patients with established/late lesions and normal controls by real-time PCR and normalized to β-actin. Data indicate individual values of cytokines in single biopsies and horizontal bars represent the median value. B. Flow cytometry analysis of IL-4-producing cells in CD3+LPMC isolated from CD patients with no endoscopic recurrence (i0–i1), CD patients with endoscopic recurrence (i2–i4), CD patients with established/late lesions and normal controls. LPMC were gated on CD3+ cells and subsequently analysed for the expression of IL-4. Data indicate individual values and horizontal bars represent the median value. Right insets: representative histograms of IL-4-producing CD3+cells in LPMC isolated from 1 CD patient with no endoscopic recurrence (i0), 1 CD patient with endoscopic recurrence (i4), 1 CD patient with established/late lesions and 1 normal control. Staining with a control IgG is also shown. Numbers above lines indicate the percentages of positive cells. C–D. Ratio between the percentages of IFN-γ-producing (C) or IL-17A-producing (D) CD3+LPMC and IL-4-producing CD3+ LPMC isolated from CD patients with no endoscopic recurrence (i0–i1), CD patients with endoscopic recurrence (i2–i4), CD patients with established/late lesions and normal controls.

Pioneering studies by Desreumaux and colleagues showed that early CD lesions are marked by enhanced gene expression of Th2 cytokines. [25] Enhanced expression of IL-4 and IL-5 was seen in CD biopsies taken from the neo-terminal ileum with endoscopic recurrence and in samples with established lesions as compared to mucosal samples taken from macroscopically unaffected neo-terminal ileum of CD patients and normal controls ( Fig. 4A–E and Tables 1 – 2 ) Analysis of the percentages of cytokine-secreting cells revealed that the fraction of IFN-γ-producing cells was 2–4 times higher than the percentage of IL-4-producing cells in CD samples (Fig. 4C). Similarly, the percentage of IL-17A-producing cells was higher than that of IL-4-producing cells in all CD subgroups, even though this difference was more marked in samples with established lesions (Fig. 4D). A more pronounced expression of IL-13 was seen in the biopsies taken from the neo-terminal ileum, either with or without endoscopic recurrence, and specimens with established lesions in comparison to control samples ( Fig. 4F and Tables 1 – 2 ).

### Early Induction of IL-12 during CD Inflammation

**Figure 5:**
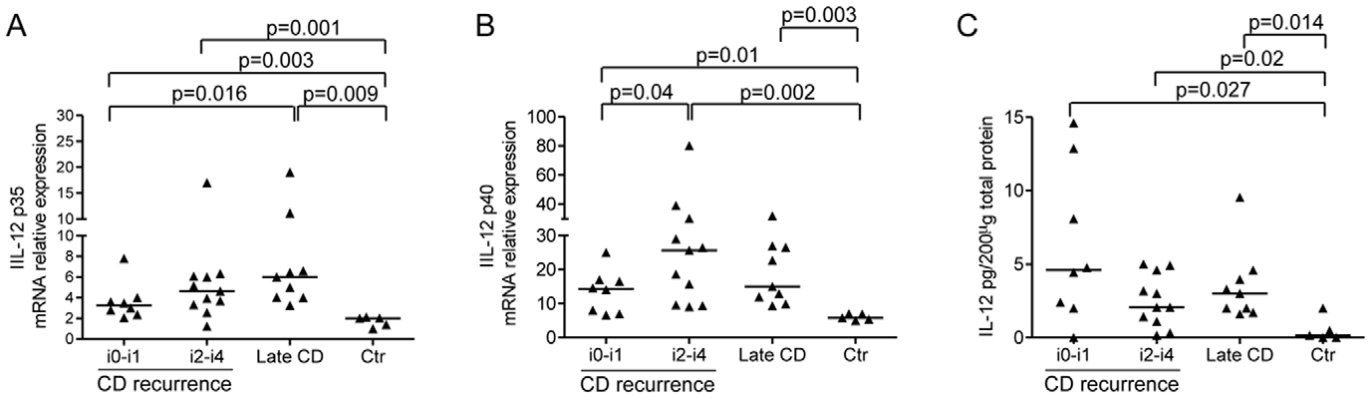
High IL-12 production in CD samples with or without macroscopically evident lesions. Transcripts for IL-12p35 (A) and IL-12p40 (B) were evaluated in ileal samples taken from CD patients with no endoscopic recurrence (i0–i1), CD patients with endoscopic recurrence (i2–i4), CD patients with established/late lesions and normal controls by real-time PCR and normalized to β-actin. Data indicate individual values of IL-12/p35 and IL-12/p40 in single biopsies and horizontal bars represent the median value. C. IL-12 heterodimer was measured in total proteins were extracted from ileal biopsies of CD patients with no endoscopic recurrence (i0–i1), CD patients with endoscopic recurrence (i2–i4), CD patients with established/late lesions and normal controls by ELISA. Data indicate individual values of IL-12 in single biopsies and horizontal bars are the median value.

Overall the above results indicate that the very early stage of CD-associated inflammation is characterized by increased expression of Th1 cytokines. As IL-12 is the major inducer of IFN-γ, [5]–[6] IL-12 RNA and protein expression was analysed in our samples by real-time PCR and ELISA respectively. RNA expression of both IL-12/p35 and IL-12/p40 subunits was more pronounced in samples taken from the neo-terminal ileum, either with or without endoscopic recurrence, and established lesions of CD patients in comparison to normal controls (Fig. 5A–B). Consistently, analysis of the heterodimeric IL-12 protein confirmed higher expression in samples of CD patients regardless of whether these were taken from areas with or without macroscopic lesions (Fig. 5C).

### IL-23, TNF and IL-6 are Differently Expressed in the Mucosa of CD Patients with Early and Established Lesions

**Figure 6:**
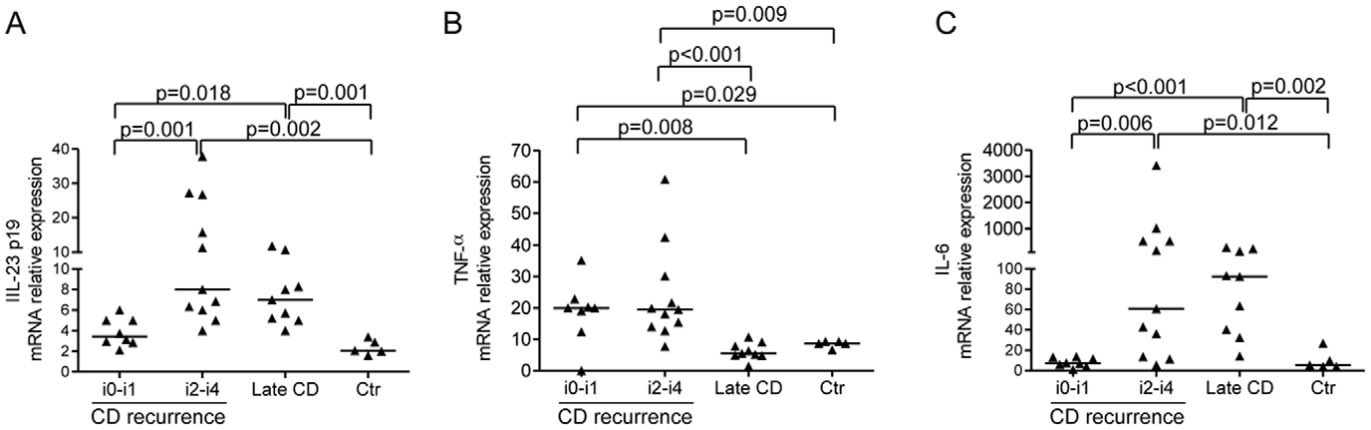
Distinct induction of IL-23, IL-6 and TNF-α in Crohn’s disease mucosa with or without lesions. Transcripts for IL-23p19 (A), TNF-α (B) and IL-6 (C) were evaluated in ileal samples taken from CD patients with no endoscopic recurrence (i0–i1), CD patients with endoscopic recurrence (i2–i4), CD patients with established/late lesions and normal controls by real-time PCR and normalized to β-actin. Data indicate individual values of the cytokines in single biopsies and horizontal bars represent the median value.

Mechanisms involved in the control of IL-17A production in humans are not fully understood, but studies performed in experimental models indicate that IL-23, TNF-α and IL-6 positively regulate IL-17A synthesis. [18], [26]–[28] The specific IL-23/p19 subunit was significantly increased in CD samples taken from the neo-terminal ileum with endoscopic recurrence and established lesions, but not from the macroscopically unaffected neo-terminal ileum, as compared to normal controls (Fig. 6A). RNA transcripts for IL-23/p19 did not significantly differ between the macroscopically unaffected neo-terminal ileum and normal controls (Fig. 6A). TNF-α was up regulated in CD samples obtained from the neo-terminal ileum, either with or without endoscopic recurrence, but not from established lesions, as compared to normal controls (Fig. 6B and Tables 1 – 2 ). IL-6 was up regulated only in CD samples obtained from the neo-terminal ileum with endoscopic recurrence and established lesions (Fig. 6C and Tables 1 – 2 ).

## Discussion

This study was undertaken to characterize the mucosal pattern of effector cytokines in CD at different stages of the disease. To this end, we considered as “initial lesions” those developing in the neo-terminal ileum of patients after a curative ileo-colonic resection and “established lesions” those seen in patients with a long-history of disease requiring intestinal resection. More than one third of CD patients did not show endoscopic signs of recurrence within the time-frame of 1 year after the ileocolonic resection, in line with previously published studies. [23], [29]–[30] Immunofluorescence analysis of biopsies taken from this subgroup of patients showed a marked infiltration of the mucosa with both CD3+ and CD68+ cells, reinforcing the notion that T cells and macrophages drive inflammatory events necessary for the development of mucosal lesions. [31]–[32].

Moreover, we found a distinct pattern of cytokines at this early stage of disease. In particular, the macroscopically unaffected neo-terminal ileum contained high levels of IFN-γ and IL-21, two cytokines which are produced by Th1 cells in humans. [5], [24] These findings are consistent with the demonstration that the macroscopically unaffected neo-terminal ileum expressed high IL-12, a strong inducer of IFN-γ and IL-21 production in the gut. [6], [33] In the same biopsies, we found a slight increase in IL-17A and elevated levels of TNF-α, a cytokine involved in the positive regulation of IL-17A synthesis [28] and supposed to play a pathogenic role in the recurrence after intestinal resection in CD. [34] In biopsies taken from areas with endoscopic lesions, expression of Th1 cytokines remained elevated and there was marked up-regulation of IL-17A and induction of IL-23 and IL-6, two cytokines which enhance IL-17A production. [26]–[27] A major strength of our study is that all patients who underwent ileocolonic resection were taking mesalamine only at the time of biopsy sampling. Thus we think it is fair to conclude that the different pattern of cytokines found in the neo-terminal ileum of CD patients with or without endoscopic lesions is not due to medical therapy.

In samples taken from mucosal areas with established lesions there were elevated levels of IFN-γ, IL-17-A, IL-4 and IL-5 as compared to normal controls. However, analysis of the cytokine expression at protein level by flow-cytometry revealed that the percentages of LPMC secreting IFN-γ or IL-17A were markedly higher than the percentage of IL-4-producing cells, reinforcing the concept that, in CD, the tissue-damaging immune response is associated with a predominant synthesis of Th1/Th17 cell-type cytokines. [1]–[2] A different Th1/Th17 cytokine ratio was however seen in the subgroups of CD patients. Indeed, the immune response in the neo-terminal ileum without endoscopic lesions was mainly polarized along the Th1 pathway while it was dominated by both Th1/Th17 cytokines in areas with either early or established lesions. These findings support previous studies in murine models of CD showing that the initial phase of the inflammation is driven by Th1 cytokines while the later phases are associated with mixed Th1/Th17 cell responses. [35]–[36] Along the same line is the Kugathasan‘s study showing that IFN-γ is over-produced in the gut of patients with CD at the first attack but not with long-standing CD. [16] Our data are however partly conflicting with those published by Kugathasan et al because we found elevated levels of IFN-γ in samples taken from patients with both early and established lesions. It is likely that this discrepancy may simply reflect differences in the methods and cell sources of cytokines used in these studies, since Kugathasan et al analysed IFN-γ in mucosal T cell clones following IL-12 stimulation while our cytokine analysis was focused on fresh biopsy and cell samples. In this context it is also noteworthy that Kugathasan’s study was performed in children and not adults and this could help explain discrepancy because it is well known that the mucosal immunological response of children may differ from that of adults [37].

Surprisingly, TNF-α was not increased in the CD mucosal specimens with established lesions, despite histopathology confirmed the severity of inflammation in all samples. If this decline in TNF-α production reflects a functional change in the immunological pathways activated during this stage of the disease or is simply secondary to the immunosuppressive therapy taken by patients remains to be ascertained.

The discovery that mucosal cytokines are temporally regulated in CD could have some potential applications that merit further investigation. For example analysis of cytokine expression at specific time points could help direct the choice of therapy and ascertain whether a patient is responding to therapy in the case the mucosal levels of cytokine change. Moreover, determining the cytokine cell sources and mechanisms involved in the control of cytokine synthesis at the different stages of the disease could provide insight into the pathophysiology of CD.

One limitation of this study is its relatively small sample size, despite a noticeable difference between CD patients and controls. However, this is the largest dataset available for patients with early CD lesions. Additionally, we should remain cautious when interpreting the physiologic implications of the Th1/Th2/Th17 imbalance in early and late CD because we analysed cytokines in whole biopsies and mucosal CD3+ T cells and not in purified CD4+ T cells. Thus, we cannot exclude the possibility that cytokines measured in our samples may derive from CD8+ T cells other than Th cells. Although we feel that prospective studies on larger numbers of patients will be needed to confirm data of this study, the cytokine expression results presented here provide evidence that there are potentially different immune mechanisms driving the early and late mucosal lesions in CD. A better understanding of such mechanisms could contribute to optimize therapeutic strategies in this disease.
